# The integrative function of a transnational policy and roadmap for action planning in implementing digital mental health

**DOI:** 10.1192/j.eurpsy.2021.61

**Published:** 2021-08-13

**Authors:** W. Gaebel

**Affiliations:** Psychiatry, LVR-Klinikum, Düsseldorf, Germany

## Abstract

**Abstract Body:**

In times of global crisis like the present Covid-19 pandemic, digital technology is rapidly conquering the health and mental health & care sector, speeding up e-Mental Health (eMH) implementation on a regional, national and global scale. Making this an organized move, guidance and regulation, legislation and training, but basically also awareness and acceptance building need to ensure the use of efficient, safe and high-quality eMH products and services. Special attention needs to focus on broadening public and professional eMH literacy, providing needs-tailored approaches for target groups, and training mental health workforce and services. Guidance, evaluation and involvement of relevant stakeholders should help to identify how citizens will best benefit from eMH&Care in its various forms. The Transnational Policy for e-Mental Health, a guidance document for European policymakers and stakeholders has been developed by the Interreg-funded eMEN project (www.nweurope.eu/emen) in six EU countries to promote implementation of high-quality eMH & care across NW-Europe. Project partners from Belgium, France, Germany, Ireland, the Netherlands and the UK contributed to product and policy-guidance development, promoting communication and research. eMEN is currently continuing its work in the Interreg-funded Capitalisation phase to scaling up the implementation of eMH&Care. The Transnational Policy within the scope of national information and training sessions on eMH will be promoted for action planning and implementation by policymakers and stakeholders at the national level. Further meetings will also take place at the European level to promote and support implementation of eMH&Care in NW-Europe and beyond.

**Disclosure:**

No significant relationships.

